# Megakaryocyte Diversity in Ontogeny, Functions and Cell-Cell Interactions

**DOI:** 10.3389/fonc.2022.840044

**Published:** 2022-02-04

**Authors:** Eman Khatib-Massalha, Simón Méndez-Ferrer

**Affiliations:** ^1^ Wellcome-Medical Research Council (MRC) Cambridge Stem Cell Institute, University of Cambridge, Cambridge, United Kingdom; ^2^ Department of Hematology, University of Cambridge, Cambridge, United Kingdom; ^3^ National Health Service Blood and Transplant, Cambridge Biomedical Campus, Cambridge, United Kingdom; ^4^ Instituto de Biomedicina de Sevilla-IBiS, Hospitales Universitarios Virgen del Rocío y Macarena/Spanish National Research Council (CSIC)/Universidad de Sevilla, Seville, Spain; ^5^ Departamento de Fisiología Médica y Biofísica, Universidad de Sevilla, Seville, Spain

**Keywords:** megakaryocyte (MK), niche, bone marrow, heterogeneity, hematopoietic stem and progenitor cell (HSPC), emperipolesis, immune

## Abstract

Hematopoietic stem cells (HSCs) rely on local interactions in the bone marrow (BM) microenvironment with stromal cells and other hematopoietic cells that facilitate their survival and proliferation, and also regulate their functions. HSCs and multipotent progenitor cells differentiate into lineage-specific progenitors that generate all blood and immune cells. Megakaryocytes (Mks) are hematopoietic cells responsible for producing blood platelets, which are essential for normal hemostasis and blood coagulation. Although the most prominent function of Mks is platelet production (thrombopoiesis), other increasingly recognized functions include HSC maintenance and host immune response. However, whether and how these diverse programs are executed by different Mk subpopulations remains poorly understood. This Perspective summarizes our current understanding of diversity in ontogeny, functions and cell-cell interactions. Cumulative evidence suggests that BM microenvironment dysfunction, partly caused by mutated Mks, can induce or alter the progression of a variety of hematologic malignancies, including myeloproliferative neoplasms (MPNs) and other disorders associated with tissue scarring (fibrosis). Therefore, as an example of the heterogeneous functions of Mks in malignant hematopoiesis, we will discuss the role of Mks in the onset and progression of BM fibrosis. In this regard, abnormal interactions between of Mks and other immune cells might directly contribute to fibrotic diseases. Overall, further understanding of megakaryopoiesis and how Mks interact with HSCs and immune cells has potential clinical implications for stem cell transplantation and other therapies for hematologic malignancies, as well as for treatments to stimulate platelet production and prevent thrombocytopenia.

## Introduction

The BM hematopoietic niches are composed of non-hematopoietic and mature hematopoietic cells that support the survival, proliferation, and differentiation of HSCs and hematopoietic progenitor cells (HPCs). HSCs and multipotent progenitor cells differentiate into lineage-specific progenitors that generate all major lineages of hematopoietic and immune cells.

HSC niches can be defined based on their anatomical location in the BM and the type of blood vessel they contain [arterioles (1), sinusoids (2), or transition zone vessels (3)]. To date, there are two anatomically and functionally distinct niches that dictate HSC cell fate in mouse BM: 1) the central niche, which is located in the inner BM, and 2) the endosteal niche, which is close to the bone surface. The central niche contains the majority of venous sinusoids and arterioles and harbours 85% of the HSCs (2). While the endosteal niche is relatively enriched in HSCs (~15% of all HSCs) (4) and contains all transition zone vessels, it seems that both BM niches are functionally different. For instance, activated HSCs migrate through the sinusoidal niche and their migration is regulated by sympathetic nerve fibers (5, 6). By contrast, the endosteal niche seems to be necessary for hematopoietic regeneration (7, 8). In line with these findings, a recent study by the Lucas group reported that granulopoiesis is spatially organized, with the generation of granulocytes and of monocytes–dendritic cells taking place in central sinusoids (9). Therefore, local cues provided by distinct microenvironments could be responsible for the orchestration of hematopoiesis.

**Figure 1:**
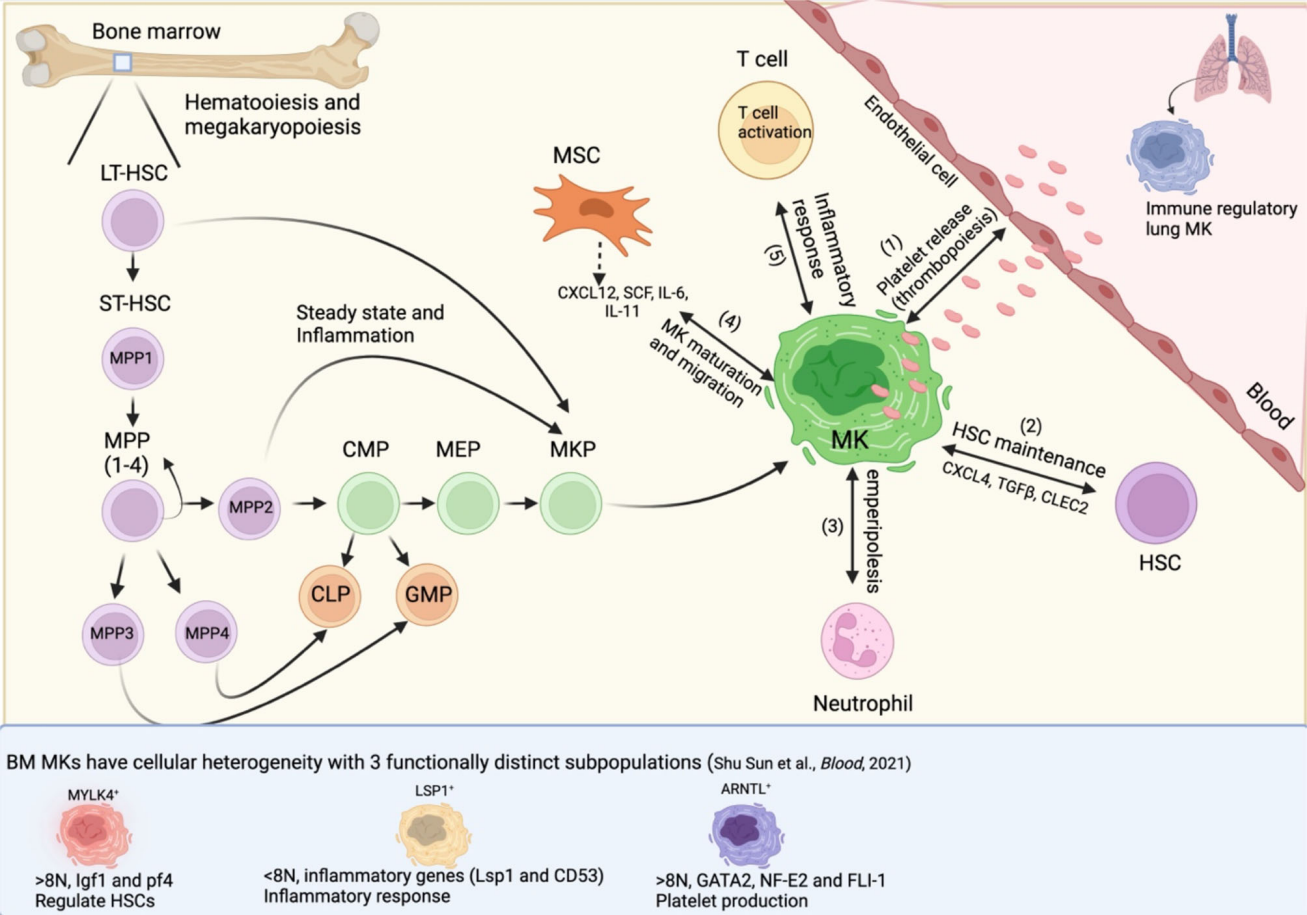
Heterogeneity in megakaryocyte development and functions. Megakaryopoiesis involves differentiation from long-term HSC (LT-HSC), short-term HSC (ST-HSC; or MPP1), multipotent progenitor (MPP2), common myeloid progenitor (CMP), Mk-erythroid progenitor (MEP), and Mk progenitor (MKP). Mks in the bone marrow undergo a maturation process involving an increase in size, extension of proplatelets and platelet release into circulation (thrombopoiesis) (1). Through divergent pathways, MPP3 differentiates into the granulocyte-macrophage progenitor (GMP), MPP4 gives rise to the common lymphoid progenitor (CLP), while MPP2 differentiates into MEP. The Mk-biased pathway is highlighted here, where MPP2 can differentiate directly into Mk bypassing intermediate progenitors, a phenomenon observed during steady-state but most frequently occurring after stress, such as inflammation. Besides platelet production and release, growing evidence supports other Mk functions, such as HSC niche cells (2). Additionally, Mks interact with other BM cells, such as neutrophils, through cellular engulfing (emperipolesis) or by regulating neutrophil migration and activation (3). In addition, Mks can interact directly with mesenchymal stem cells (MSCs) *via* adhesion molecules, while MSCs secrete cytokines, chemokines and soluble factors that affect Mk maturation and migration (4). New studies have shown that Mk can also act as inflammatory cells and enhance CD4^+^ T cell activation and function (both in BM and lung). BM Mks might comprise 3 functionally distinct subpopulations that can be identified using different markers: Myosin Light Chain Kinase Family Member 4 (MYLK4)^+^ Mks involved in regulating HSC maintenance; Lymphocyte Specific Protein 1 (LSP1)^+^ inflammatory response-associated Mks; and platelet-releasing Aryl hydrocarbon receptor nuclear translocator-like protein 1 (ARNTL)^+^ Mks (16).

Megakaryopoiesis is the process giving rise to Mks within the myeloid branch of hematopoiesis. During steady-state hematopoiesis, all blood lineages are produced through a series of committed progenitors, the Mk being derived through the multipotent progenitor (MPP), common myeloid progenitor (CMP), and Mk erythroid progenitor (MEP) (10–12); however, recent studies have begun to redefine this hierarchy and shed new light on alternative routes by which HSCs can directly differentiate into Mks (13–15) (**Figure 1**).

Mks are one of the largest (50-100 μm) and rarest (0.05% to 0.1%) hematopoietic cells in the BM. Mk progenitors undergo multiple rounds of endomitosis to become polyploid cells (17, 18). Polyploid Mks further undergo terminal maturation and generate platelets, essential for normal hemostasis and blood coagulation. Although the most prominent function of Mks is the production and release of platelets (19), growing evidence attributes new functions to these cells in the generation and maintenance of HSCs, an effect mediated by cytokines and growth factors such as CXCL4 (PF4), TGF-β, FGF-1, and IGF-1 (20–24). In addition, recent studies have reported that Mks may participate in regulating the immune response during inflammation and infection because they express multiple inflammatory and immunologic surface markers. Thus, Mks can be considered immune cells, as well as hemostatic cells (25–27).

Furthermore, Mks are the primary source of pro- and anti-angiogenic proteins (e.g. VEGF, Thrombospondin-1 and Endostatin) (28) and the “profibrotic” protein TGF-β, which is involved in the onset and progression of MPNs (29, 30). All these findings raise the question of whether these distinct Mk functions are executed by the same cells or by different subsets of cells. This Perspective article focuses on recent studies that have expanded the functions of Mks beyond thrombopoiesis and shed light on Mk heterogeneity, Mk interactions with other BM cell types, and Mk functions under physiological and pathological conditions (particularly in MPN pathogenesis).

## Megakaryocyte Development and Heterogeneity

BM long-term HSCs (LT-HSCs) are largely quiescent during steady-state hematopoiesis (31). However, during emergency hematopoiesis, HSCs lose quiescence, differentiate into mature hematopoietic and immune cells and are mobilized into circulation. Hematopoiesis is a stepwise differentiation process from LT-HSCs, short-term HSCs (which can reconstitute all mature lineages but have limited self-renewal capability) and MPPs, which are non-self-renewing lineage-biased progenitor cells. MPPs differentiate into committed progenitors, such as common lymphoid progenitors (CLP) and CMPs, which differentiate into MEPs and Mk progenitor (MKPs) (**Figure 1**).

The traditional view of megakaryopoiesis describes the progressive commitment from hematopoietic stem cells, through a stepwise cascade of differentiation, into mature Mks. At the end stage, Mks undergo a terminal maturation process involving multiple steps of endomitosis and cytoplasmic restructuring to form platelets, which is the main function of Mks. However, recent studies have challenged this model of hematopoiesis and proposed alternative routes by which HSCs can directly differentiate into Mks (15).

As platelets play a role in inflammation, infection and vascular injury, it is expected that an alternative, faster megakaryopoiesis mechanism skipping intermediate steps is available. During stress megakaryopoiesis, rapid differentiation from HSCs might be required to replenish the platelet pool. It is important to note that the MPP population comprises subsets of lineage-biased MPPs (32), which have been designated MPP1–4 (33). Integrated quantitative proteome, transcriptome, and methylome analyses of these four MPP populations showed that MPP1 resemble short-term HSC, whereas MPP2 are biased towards Mks/erythroid cells, MPP3 are granulocyte/macrophage biased, and MPP4 are lymphoid biased (33) (**Figure 1**). Importantly, MPP2 seems to be the only MPP capable of replenishing platelets in a transplant model (32).

Along this line, a recent study has confirmed the existence of Mk-biased MPP2 and HSCs responsible for the generation of ~31% Mks during native hematopoiesis (15). Notably, mature Mks can differentiate directly from LT-HSCs by skipping the MPP2 intermediate (**Figure 1**). Taken together, these critical studies support the idea that Mk-biased HSCs are important to directly and rapidly differentiate into Mks, bypassing intermediate steps (like CMP or MEP). The above-mentioned studies also suggest that HSCs might originate as Mk-primed during development (13, 14, 34).

Mks have been found associated with vascular BM niches as they are frequently adjacent to BM sinusoids (35, 36). However, Mks can locate close to different types of BM vessels, such as venous sinusoids and arterioles (37), in the endosteal or central BM. Notably, it is known that positioning of Mk in close proximity of BM sinusoids relies on chemokines that are produced and released by endothelial cells and mesenchymal stromal cells (MSCs) associated with these vessels (38, 39). An investigation of the BM of mice two days after sub-lethal total body irradiation revealed that Mks can translocate from the epiphysis to the diaphysis in response to SDF1 (CXCL12) upregulation in these areas upon BM injury (39, 40).

**What are the signaling pathways that regulate Mk development?** Megakarypoiesis is regulated at multiple levels by different cytokines, with the most critical one being thrombopoietin (TPO). In 1994, TPO and its receptor, c-Mpl, were cloned and found to stimulate platelet production (41, 42). Several studies showed the same year that TPO signaling promotes both development and maturation of Mks from HSCs (41–46). Correspondingly, all Mk-competent or primed progenitor cells, including HSCs, CMPs and MEPs, express Mpl (47). Consequently, mutations in c-Mpl or TPO can cause congenital amegakaryocytic thrombocytopenia (CAMT), which is characterized by severely defective megakaryopoiesis (48–54).

TPO signaling results in the internalization of the Mpl-TPO complex and the initiation of signal transduction pathways. TPO induces phosphorylation of JAK2, which in turn phosphorylates and activates downstream targets, including the transcription factors STAT3 and STAT5 (55) (this specific pathway and its involvement in MPN pathogenesis is discussed in further detail below). Finally, signaling through these pathways causes downstream activation of Mk-specific transcription factors. To emphasize the role of TPO in HSC and Mk production, the group of Alexander used TPO and Mpl knock-out (KO) mice and found that these mice have decreased HSCs, leading to a significant reduction in Mks and platelets. Taken together, these data imply that TPO signaling through Mpl receptor plays a vital physiological role in the regulation of HSC production and function (56).

In 2007, the Suda group reported that TPO/Mpl signaling regulates HSC quiescence and mobilization by upregulating beta1-integrin and cyclin-dependent kinase inhibitors in HSCs (57).

Later, in 2015, the same group identified CLEC-2, a novel Mk-specific factor involved in TPO production in Mks. In this study, the authors demonstrated that Mk-specific deficiency of CLEC-2 disrupts HSC quiescence and that CLEC-2 crosstalks with other niche pathways, including TPO, in Mks (58). Indeed, TPO is essential for the rapid Mk commitment of platelet-biased HSCs. TPO-dependent changes in mitochondrial metabolism prime HSCs to undergo direct differentiation into Mks, while skipping intermediate progenitors (59).

Besides TPO, several other cytokines and chemokines have been identified as Mk-promoting factors. For example, a recent study showed that Insulin-like growth factor (IGF-1) can promote CD34^+^ cell differentiation toward Mks, as well as facilitate pro-platelet formation and platelet production from cultured Mks – a process mediated by AKT signaling, with the assistance of steroid receptor coactivator 3 (60). Additionally, the CCL5 chemokine has been shown to increase Mk ploidy and pro-platelet formation in a CCR5-dependent manner (61). In this study, using an *in vivo* murine acute colitis model, the authors found that platelet count significantly correlates with inflammation, whereas CCR5 antagonist treatment abolishes this correlation. Therefore, CCL5/CCR5 signaling may increase platelet counts during physiological stress.

In addition to IGF-1 and CCL5, several members of the IL-6 family, including IL-6, IL-11 and leukemia inhibitory factor (LIF), have a major role in Mk maturation (62, 63). However, genetic ablation of these cytokines or their specific receptor had no essential part in normal steady-state megakaryopoiesis and are not required for the residual Mk and platelet production found in the *Mpl^-/-^* mouse model (64, 65).

To conclude, excepting TPO, the cytokines discussed above, while efficiently stimulating megakaryopoiesis *in-vitro* or *in-vivo*, appear to be dispensable when ablated in genetically modified mice. These observations, combined with the fact that many of these cytokines are produced and released in high levels by different immune cells in response to viral/bacterial infection or inflammation, indicate that these cytokines might be important to drive emergency (rather than steady-state) thrombopoiesis.

As mentioned before, Mks have other functions different from platelet production. Mks can serve as HSC niche cells and regulate HSC function by secreting cytokines such as CXCL4 (PF4), TGF-β, FGF-1, and IGF-1 (20–23). Furthermore, Mks may participate in pathogen surveillance and antigen presentation because they express multiple inflammatory and immunologic surface markers, including members of the toll-like receptor (TLR) family (TLR1-6), FcγR, major histocompatibility complex (MHC) class I, and CD40L (25–27). In addition, Mks have anti-viral functions since they produce interferon (IFN)-induced transmembrane protein 3 (IFITM3) to limit viral infection (66). Mks also produce and release keratinocyte chemoattractant (KC or CXCL1), which promotes neutrophil motility and mobilization from the BM (67). However, it is not known whether these diverse programs are executed by a functionally homogeneous population or by distinct subsets of Mks. In other words, the functional heterogeneity among Mks remains unclear, partly due to the difficulty to isolate Mks due to their size and complexity.

However, a very recent study by Sun and colleagues (16) has combined improved isolation methods with scRNA-seq analysis, *in situ* 3D immunofluorescence and functional assays, to characterize the functional diversity and spatial distribution of Mks. The authors identified four subpopulations/clusters associated with unique functions, including platelet generation, maintenance of the HSC niche, and inflammatory responses (**Figure 1**). Importantly, these transcriptionally distinct groups of Mks were similarly identified in primary human BM Mks. The role of each subpopulation is discussed in further detail below.

## Megakaryocyte Interaction With Different Bone Marrow Niches

Mks interact with different hematopoietic and stromal cellular components while migrating between endosteal and central niches within the BM. This section focuses on Mk-BM cell interactions and how these affect Mk development, maturation, and function.

Cumulative evidence indicates that MSCs are key regulators of Mk differentiation and function (68),. MSCs express and secrete different important regulators of Mk maturation, such as, IL-6, IL-11, and Stem Cell Factor (SCF) (**Figure 1**) (63, 69). In addition to secreting cytokines, MSCs directly interact with Mks through cell adhesion molecules like ICAM-1, VCAM-1 and E-Selectin (70). Furthermore, SDF-1 (CXCL12) secreted by MSCs can direct the migration of Mks, which express the CXCL12 receptor CXCR4 (**Figure 1**). CXCL12 is mainly produced in the BM by perivascular MSCs and, to a lesser extent, by endothelial cells (71, 72).

Apart from MSCs, endothelial cells can regulate Mk differentiation and thrombopoiesis (73, 74). BM endothelial cell monolayers can support long-term proliferation of HPCs, particularly megakaryocytic and myeloid progenitor cells, through the secretion of cytokines such as G-CSF, GM-CSF and IL-6. Direct cellular contact between HPCs, Mks and BM endothelial cells through specific adhesion molecules, including β1 and β2 integrins, plays a critical role in the migration and possibly in the proliferation of HSCs. However, dysfunction of endothelial cells within the hematopoietic microenvironment [e.g. during inflammation, hypoxia or exposure to damaging stimuli (75, 76)], which is characterized by higher levels of reactive oxygen species (ROS) and apoptosis, impaired migration and angiogenesis, may contribute to the pathogenesis of aplastic anemias and contribute to graft failure after BM transplantation (77–79).

Under pathological conditions (e.g. MPNs), the Zhan group used mice expressing the *JAK2^V617F^* mutation specifically in ECs (found in some MPN patients; using *Tie2-Cre*), to study the effect of the *JAK2^V617F^*-bearing vascular niche on MPN disease development *in vivo* (80). The authors observed thrombocytosis and clusters of Mks preferentially located near sinusoids, associated with reticulin fibrosis. Since Mks play an important role in the pathogenesis of marrow fibrosis (29), these findings suggest that the *JAK2^V617F^* mutation might have direct fibrotic effects on mutant Mks, but also niche-dependent effects, due to the impact of the mutation in ECs. The role of Mk-derived profibrotic cytokines is discussed in further detail below.

Bone turnover and remodeling is finely regulated by the concerted action of bone-resorbing osteoclasts and bone-forming osteoblasts. Increased bone resorption and/or decreased bone formation can reduce bone mass and quality, resulting in high fracture risk. Increasing *in vitro* and *in vivo* evidence suggest that Mks regulate both bone resorption and bone formation. A mouse model with increased Mks showed a high bone mass phenotype and decreased osteoclast number and bone resorption (81). *In vitro* experiments have demonstrated that Mks inhibit osteoclast precursors from differentiating into osteoclasts, suppressing bone resorption (82). In contrast, Mks promote osteoblast proliferation and bone formation (83). Thus, Mks play an osteoprotective role by inhibiting bone resorption and stimulating bone formation, making it an ideal therapeutic target.

To test the roles of Mk-secreted factors on bone formation and resorption, a recent study by Lee et al. found that Mk-conditioned media reduced *in vitro* bone resorption due to suppressed osteoclastic activity (84). Mk-conditioned media suppresses osteoblastic differentiation but stimulates osteoblast proliferation, prompting bone formation. While Mks have bone-anabolic effects, little is known about the regulation of megakaryopoiesis and platelet formation by osteoblasts. A recent study has suggested that osteoblasts support megakaryopoiesis by secreting interleukin-9 (IL-9), which stimulates IL-9 receptor (IL-9R)/STAT3 signaling to drive megakaryopoiesis (85).

## Emperipolesis: An Abnormal Megakaryocyte -Neutrophil Interaction

Mks have long been known to internalize other intact hematopoietic cells, but the pathophysiological implications of these observations have remained unclear. Already in 1970, while studying fresh BM samples from thrombocytopenic and anemic patients, Larsen et al. noted neutrophils invading and moving inside the demarcation membrane system of Mks – the cytoplasmic network that forms the plasma membrane of future platelets – enabling the transfer of membrane from the neutrophil to the Mks and vice versa, and later exiting, without compromised cell viability (86). This phenomenon was termed “**emperipolesis”**. Since then, BM Mk emperipolesis has been observed in a number of pathological conditions, where neutrophils are usually the most frequently internalized cells. More than 50 years since its initial observation and description, the cell biology, regulation, and function of Mk emperipolesis remain largely unknown.

Emperipolesis occurs at low frequency in the healthy BM and may increase across a range of pathological conditions. Both *in vitro* modeling and *in vivo* studies have established that emperipolesis is a distinctive form of cell-cell interaction different from phagocytosis, entosis, or transcellular migration (87). Studies focused on the interaction between Mks and neutrophils underscore the need of active cytoskeletal engagement by both cell types for emperipolesis. Ultrastructure (electron microscopy) and immunofluorescence studies have suggested a multi-step process: First, neutrophil enter Mks in vesicles termed “emperisomes”, which are larger than the engulfed cells, but contract over time. Next, the emperisome membrane disappears, leaving the intact neutrophil in the demarcation membrane system of Mks. After a period of residence, neutrophils exit the host Mk without evident damage to either cell type (87, 88).

Emperipolesis is likely mediated by specific receptor-ligand interactions. Tanaka M et al. found enhanced intracellular adhesion molecule 1 (ICAM-1) staining in Mks engaged in emperipolesis (89). At the same time, internalized neutrophils expressed the lymphocyte function-associated antigen1 (LFA-1, or CD18/CD11a), which is the receptor for ICAM-1. Consequently, LFA-1 blockade reduces lipopolysaccharide (LPS)-induced emperipolesis *in vivo* (89).

In 2000 Cramer and colleagues made the observation that Mks from primary myelofibrosis (PMF) patients and mouse models highly express the adhesion molecule P-selectin on their demarcation membrane system, associated with very frequent emperipolesis (90). Studies by the Miggliacio group supported this concept by demonstrating that impaired Mk maturation in the *GATA1^low^* animal model of myelofibrosis leads to abnormal P-Selectin distribution and emperipolesis (91), raising the possibility that neutrophil proteases might facilitate the pathological release/activation of Mk α-granule proteins and growth factors, such as TGF-β, which are critical in the pathogenesis of myelofibrosis (92). However, Mks from PMF patients and mouse models exhibit numerous maturation abnormalities. Consequently, whether abnormal P-selectin in the DMS-mediated neutrophil emperipolesis within Mk is solely responsible for TGF-β-induced myelofibrosis remains to be demonstrated.

These abnormal Mk-neutrophil interactions raise the question whether Mks regulate neutrophil activation, migration or function. Mk cytoplasm and α-granules are rich in soluble mediators that can impact immune cells, and particularly neutrophils [reviewed in (93)]. For instance, platelet factor 4 (PF4), TGF-β, and CXCL1 are produced by Mks and promote neutrophil motility and mobilization from the BM (67, 94, 95). Mks and platelets also produce pro-inflammatory cytokines considered as potent neutrophil activators, including IL-1α, IL-1β and IL-6 (93, 96, 97). Thus, emperipolesis likely modulates the function of neutrophils transiting inside Mks, which in turn affect the function of Mks and platelets. However, further investigations are warranted to elucidate the molecular underpinning and functional implications of Mk-neutrophil emperipolesis in myelofibrosis.

Mk-neutrophil emperipolesis is frequently observed in other types of myelofibrosis independent from MPN, such as in those associated to Mk genetic disorders, such as Gray Platelet Syndrome (GPS). GPS is a rare inherited bleeding disorder characterized by a deficiency of platelet α-granules, macro-thrombocytopenia and BM fibrosis. The autosomal recessive form of GPS is linked to loss of function mutations in *NBEAL2*, which is predicted to regulate granule trafficking in Mks. Unexpectedly, the Guerrero and Ouwehand group observed that GPS platelets are enriched in proteins normally found within neutrophil granules (98). Due to the lack of concordant increase in expression of the genes encoding these proteins, two potential mechanisms are: 1) endocytosis by circulating platelets or 2) emperipolesis by Mks. Although we cannot discard the former, we and others hypothesize that emperipolesis likely affects the cargo of platelets. A transfer of membranes between Mks and neutrophils has been observed in emperipolesis (87), and a functional genomic study reported an association of myeloperoxidase (MPO, normally contained within neutrophil-specific granules) with mean platelet volume, highlighting the crosstalk between neutrophils and platelets (99). Hence, the release of neutrophil granule proteins (such proteolytic enzymes, elastase and MPO) inside Mks during emperipolesis could provide a plausible explanation for the higher level of these proteins in GPS platelets (98).

## Megakaryocytes as HSC Niche Cells

In both central and endosteal BM, HSCs reside in perivascular niches, where endothelial cells and MSCs critically maintain and regulate HSC function. Pioneering studies by the Nilsson group showed that transplanted HSCs preferentially lodge close to mature Mks in the mouse BM and that HSCs isolated from the endosteal BM proliferate more when co-cultured with mature Mks (22). However, *in vivo* studies suggest that Mks preserve HSC quiescence; whole-mount 3D BM imaging revealed that a subset of quiescent HSCs specifically associates with Mks (20). In addition, Mks, which are mainly adjacent to sinusoids, regulate HSC quiescence by several mechanisms under homeostatic conditions, including secretion of the chemokine CXCL4/PF4 (20) or TGF-β (23, 100). Furthermore, Mks can also regulate HSC quiescence through C-type lectin-like receptor-2 (CLEC-2) signaling, since *Clec2MkΔ/Δ* mice exhibit reduced BM HSC quiescence and repopulation potential, along with extramedullary hematopoiesis. The low level of TPO production may account for the decline in HSC potential in *Clec2MkΔ/Δ* mice, as administration of recombinant TPO to *Clec2MkΔ/Δ* mice restores HSC potential. Overall, this study shows that CLEC-2 signaling is involved in various molecular pathways to produce niche factors, including TPO (58). Taken together, these studies reinforce the idea that Mks function as a niche to maintain HSC quiescence. However, under stressed conditions, when rapid expansion of HSCs is required, Mks produce fibroblast growth factor-1 (FGF-1), promoting HSC expansion after injury (23, 24). In addition to the direct release of proteins, recent attention has also turned to the release of microparticles within the BM. Mk-derived microparticles bind to HSPCs and induce them to differentiate into functional Mks (101), providing a possible mechanism of direct communication between the Mk and the HSC.

The spatial localization of HSCs in the BM remains controversial, with some studies suggesting that they are maintained in homogeneously distributed niches, while others have suggested the contributions of distinct niche structures. HSC quiescence may be differently regulated between steady-state and emergency and/or malignant hematopoiesis. However, lineage commitment appears to be influenced by the location of HSCs in the BM. Growing evidence suggests that lymphopoiesis preferentially occurs in the endosteal BM, while myelopoiesis/erythropoiesis/megakaryopoiesis mainly occurs in central BM regions. A study by the Frenette group supported this concept by using von Willebrand factor (vWF)-eGFP mice model to label platelet-biased HSCs (102). In this study, the authors demonstrated that platelet and myeloid-biased HSCs, marked by vWF expression (vWF^+^ HSCs), are highly enriched near MKs. Moreover, they found that depletion of Mk selectively expands vWF^+^ HSCs, whereas the depletion of NG2^+^ arteriolar niche cells selectively depletes vWF^-^ lymphoid-biased/balanced HSCs (102). In other words, vWF+ platelet/myeloid-biased HSCs are associated with Mks, whereas vWF- lymphoid-biased/balanced HSCs are located close to arterioles. Therefore, alterations in specialized niches might directly affect myeloid/lymphoid output, and the imbalanced production of mature hematopoietic cells at specific niches might in turn, remodel the local microenvironment for these cells.

As described above, Mks are known to play critical roles in supporting HSCs in the BM niche. Recently, Sun et al. identified a transcriptionally unique Mk subpopulation that expresses high levels of Mylk4 (cluster 2; HSC-niche MKs) (16). These Mks highly express other HSC regulators, such as CXCL4, IGF1, WNT signaling and cell adhesion molecules. The unique spatial distributions of cluster 2 (HSC niche Mks) and cluster 4 (platelet generation Mks) populations were consistent with the distinct function inferred from their respective transcriptomic signatures and support the concept that platelet generation and HSC maintenance are properties of 2 different Mk subpopulations. Additionally, these observations provide the foundation for future studies to investigate the interaction between Mks and HSCs.

Mks promote the quiescence of neighboring HSCs. Nonetheless, whether Mk-HSC interactions change during pathological conditions or during aging is unclear. Sympathetic adrenergic signals regulate stromal MSC proliferation (71) and are affected during age-related myeloproliferative neoplasms (103). Cumulative evidence suggests that different BM microenvironments regulate myeloid differentiation and megakaryopoiesis during aging (104). One study suggests that the BM microenvironment regulates HSPC lineage commitment through β_2_-adrenergic-receptor (β_2_-AR) and β_3_-AR. Interestingly, β_2_-AR and β_3_-AR exhibit opposite roles on myeloid differentiation: increased BM noradrenergic innervation promotes β_2_-AR-IL-6-dependent megakaryopoiesis and becomes predominant during aging, while reduced β_3_-AR activity correlates with decreased endosteal niches and Mk apposition to sinusoids (104). Additionally, increased sympathetic adrenergic activity has been previously described during aging (105) and might increase osteoporosis and fracture risk by restraining bone formation (106).

## Megakaryocytes and Their Important Roles in Immunity

Emerging evidence demonstrates that Mks sense and respond to inflammatory stress and participate in host immune responses. For instance, infections are associated with extensive platelet consumption, representing a high risk to health. However, the mechanism coordinating the rapid regeneration of the platelet pool during such stress conditions is not entirely understood. Haas S et al. reported that the phenotypic HSC compartment contains stem-like Mk-committed progenitors (SL-MkPs) (107). This cell population shares many features with multipotent HSCs and serves as a lineage-restricted emergency pool. Inflammatory signaling associated with infections directly triggers cell cycle activation of quiescent SL-MkPs, resulting in rapid maturation of SL-MkPs and other Mk progenitors, thereby efficiently replenishing platelets lost during acute inflammation (107).

Under stress conditions, such as inflammation and infection, murine Mks express MHC class II genes, which are predominant markers of professional antigen-presenting cells, such as dendritic cells, macrophages, and B cells, and co-stimulatory molecule CD40L – the ligand for the CD40 on T cells and B cells. However, whether the expression of these immune-related genes is ubiquitous in the Mk lineage or restricted to a subset of Mk populations *in vivo* is unknown. Using freshly isolated mouse cells, Sun et al. found that inflammatory CD53^+^ Mks are capable of engulfing and ingesting bacteria particles, whereas CD53^–^ Mks lack phagocytic capability (16). Moreover, when co-cultured with T cells, inflammatory CD53^+^ Mks, but not CD53^–^ Mks, can stimulate T-cell expansion. Together, these observations indicate that CD53^+^ Mks can respond to pathogen-derived agonists and inflammatory stimulation. Therefore, the CD53^+^ Mk subset appears to have specialized functions in innate and adaptive immunity.

Interestingly, in a systemic lupus erythematosus mouse model, Lin^–^c-Kit^+^CD41^+^ Mk progenitors express MHC class II molecules and can promote Th17 cell development *in vitro* (108). In line with this result, Mks and platelets are also involved in induction of immune tolerance as they can control the differentiation of Th17 and the expression of Foxp3^+^ Treg cells (109, 110). Taken together, these findings suggest that Mks have dual functions, as they may serve as antigen-presenting cells through MHC class II molecules for platelet-mediated immunoregulation while they might also undergo active thrombopoiesis to replenish the pool of circulating platelets during emergency thrombopoiesis, such as after bleeding (108, 111). However, it is currently unclear whether Mks with immune functions undergo thrombopoiesis, or whether the resulting platelets are functionally different from those derived from other Mk populations.

Platelet-derived CD40L was shown to induce monocyte differentiation into dendritic cell (DC), DC maturation and upregulation of co-stimulatory molecules (112). This function of CD40L derived by platelets may be highly relevant to autoimmune diseases, such as systemic lupus erythematosus, in which platelets induce DC differentiation and type-I interferon release, thereby promoting B-cell secretion of antibodies (113).

In addition to CD40L, Mks also express CD80 and CD86 lymphocyte co-stimulation molecules (114). This study demonstrates that Mks can endocytose ovalbumin and proteolytically generate its immunogenic peptide ligand, which is cross-presented on their surface in association with MHC class I. The authors also found that MKs can present Mk-associated (CD61) peptides to activate CD61-specific CD8^+^ T cells and mediate immune thrombocytopenia *in vivo*. Together, these results suggest that, in addition to their hemostatic role, mature Mks can significantly affect CD8^+^ T-cell responses *via* antigen presentation and are able to spread this immunogenic information through platelets. Interestingly, a recent study by the Ziebuhr group identified an expansion of interferon-activated circulating Mks and increased erythropoiesis in patients with COVID-19 (115). Mk- and erythroid-cell-derived co-expression modules were predictive of fatal disease outcome. The study demonstrates broad cellular effects of SARS-CoV-2 infection beyond adaptive immune cells.

In summary, Mks can play vital roles in adaptive immunity and during infections. In addition to their support of professional antigen-presenting cells and lymphocyte functions, Mks can process and present antigens.

## Megakaryocytes Outside the Bone Marrow

Mks have long-been considered BM-resident cells. However, platelet-producing Mks can be found in other tissues, such as lung interstitial tissue (116), although these cells could be derived from the BM and their contribution to physiological thrombopoiesis is unclear. Compared with BM, lung interstitial Mks are enriched with mRNAs associated with immune regulatory functions, such as immunoreceptors, chemokines, and cytokines (116). The role of Mks as immune regulatory cells is poorly understood in general, particularly regarding lung interstitial Mks. Since these cells increase during pulmonary and cardiovascular diseases (117), lung interstitial Mks may be dynamically responsive to inflammatory states.

Mks have been mainly studied in the immune-quiescent BM environment, and it is unknown whether Mks in other tissues have a different immune phenotype and functions. Recently, Pariser et al. have demonstrated that lung and BM Mks have distinct immune phenotypes and functions: lung Mks secrete inflammatory cytokines and express molecules that are similar to many tissue-resident leukocytes and antigen-presenting cells (118, 119). Moreover, lung Mks process live intact bacteria and present bacteria-derived antigen to CD4^+^ T cells both *in vitro* and *in vivo*. This study suggests that lung Mks have important roles in the early activation of T cell responses to pulmonary pathogens, supporting an immune regulatory function of lung interstitial Mks (**Figure 1**) (118, 119). Sun et al. compared the identified four BM MK clusters with lung Mks (16). BM immune Mks are preferentially enriched in the expression of antigen presentation-related genes. In contrast, lung immune Mks exhibit stronger expression signatures of phagocytosis and bacteria clearance, which is consistent with the notion that lungs face more constant challenges from pathogens, compared with BM.

## Megakaryocyte Dysfunction in Hematological Malignancies, Including MPNs

The past few years have seen extraordinary support for the dysfunction of the BM microenvironment, affecting BM Mks, in the progression of a variety of hematologic malignancies, including MPNs (120). MPNs are clonal hematopoietic disorders characterized by excessive production of mature blood cells. The Philadelphia-negative MPNs include three major diseases: polycythemia vera (PV), essential thrombocythemia (ET), and primary myelofibrosis (PMF) (121). Several mutations affecting the genes encoding* *JAK2, CALR,* *or* *MPL, referred to herein as the “oncogenic drivers”, are considered to drive the myeloproliferative phenotype. These mutations lead to constitutive activation of cytokine-regulated intracellular signaling pathways (122, 123). The last two decades have spotted dramatic advances in understanding of the molecular and cellular basis of excessive myeloproliferation, leading to the development and therapeutic use of novel targeted treatments, such as JAK inhibitors. In this section, the role of Mks in the onset and progression of MPNs and MPN-related myelofibrosis, will be discussed.

Myelofibrosis can occur as a primary disorder (PMF) or develop secondary to the other MPN classifications (PV or ET). PMF is the prototypic example of progressive development of BM fibrosis and is associated with poor prognosis and morbidity. In addition, myelofibrosis is characterized by increased numbers of abnormal BM Mks, and progressive BM fibrosis that destroys the hematopoietic microenvironment, resulting in the cardinal disease features of cytopenias, mobilization of HSPCs to peripheral blood, extramedullary hematopoiesis, splenomegaly, and a high propensity for leukemic transformation. Survival is typically 5–10 years from diagnosis and is not substantially improved by currently available drug therapies (124).

Mks are dramatically increased in number in myelofibrosis and are one of the critical cellular drivers of the destructive BM remodelling by releasing excess pro-fibrotic growth factors, cytokines including TGF-β, cross-linking enzymes and extracellular matrix proteins (28, 125–128). All these proteins are known to directly cause BM fibrosis by stimulating MSCs to become fibroblastic cells or produce collagen, leading to ECM remodeling and BM scarring. An alternative source of collagen- and fibronectin-producing cells in the BM and spleens of JAK2^V617F^-induced myelofibrosis mice are fibrocytes derived from neoplastic hematopoietic cells (mainly from neoplastic monocytes) upon TGF-β stimulation (129).

TGF-β is a member of a large family of growth factors involved in tissue development and repair, as well as in cancer progression and myelofibrosis. Using transmission electron-microscopy, the Migliaccio group identified Mks with abnormally high levels of TGF-β and collagen fibres embedded in their cytoplasm in the spleen of PMF patients and *Gata1low* mouse model of PMF (130). Importantly, TGF-β inhibition or loss of P-selectin (involved in Mk-neutrophil interaction) could prevent fibrosis in this study.

The cellular and molecular pathways that give rise to the dramatically increased Mk numbers and Mk dysfunction leading to tissue fibrosis are unclear. Recently, the Mead group investigated the cellular and molecular basis for aberrant megakaryopoiesis in myelofibrosis (131). In this study, the authors captured peripheral blood HSPCs and performed single-cell analysis of abnormal Mk differentiation and function in patients with myelofibrosis. Using multi-parameter immunophenotyping, functional studies, single-cell RNA sequencing, TARGET-seq (132), and single-cell proteomics, they found a fibrosis signature already at the Mk progenitor stage in myelofibrosis (PMF, post ET myelofibrosis, post PV myelofibrosis), identifying potential targets for future studies (131). Among these was G6B, an immunoreceptor tyrosine-based inhibition motif (ITIM), a Mk marker over-expressed in mutant clone-derived HSPCs from myelofibrosis patients (131). These investigators propose the combinatorial targeting of HSCs and Mks with bi-specific antibody therapies as a potential future strategy for selective ablation of the myelofibrosis clone.

Although the molecular and cellular mutations involved in the pathogenesis of MPN and PMF have been extensively investigated (133), reactive cellular alterations in the non-hematopoietic compartment (mainly in stromal cells) remain comparatively less clear. For instance, a recent study by Baryawno et al. found that different stromal subtypes have distinct roles in normal BM hematopoiesis and leukemia (134). However, the sequential events that drive stromal cell activation by hematopoietic–stromal crosstalk remain elusive. Recently, through an unbiased approach and validation in patients with MPN, the Schneider group found that the differential spatial expression of the chemokine CXCL4 might trigger the progression to fibrosis (135). In this study, the authors show that the absence of hematopoietic CXCL4 ameliorates the MPN phenotype, reduces stromal cell activation and BM fibrosis, which in turn decreases the activation of profibrotic pathways in Mks, inflammation in fibrosis-driving cells, and JAK activation in both Mks and stromal cells in three murine PMF models (TPO^high^, JAK2^V617F^, MPL^W515L^). These data indicate that excessive CXCL4 levels, associated with the characteristically high inflammation in MPN, have pro-fibrotic effects. Therefore, targeting CXCL4 might be a promising strategy to reduce inflammation and fibrosis in PMF.

Patients with BM fibrosis have increased levels of extracellular matrix (ECM) proteins, particularly reticulin, fibronectin fibers and, in some cases, collagen fibers. Mks are presumed to be the neoplastic cell subtype that predominantly forces fibroblasts to produce ECMs in the disease, through an uncontrolled production and release of several cytokines, such as TGF-β1, platelet-derived growth factor, or basic fibroblast growth factor [as reviewed in (136)]. Among the ECM proteins that fill the BM cavity, fibronectin is a glycoprotein whose mRNA has three alternative splicing sites (termed extra domain A [EDA], extra domain B [EDB], and IIICS or EIIIA, EIIIB, and V), that allow several forms of fibronectin, whose expression has been proven to be altered in tumors (137). Different studies have described the involvement of EDA fibronectin isoforms in critical pathological processes, such as atherosclerosis (138), lung fibrosis (139), and liver fibrosis (140). On the contrary, its expression and function in BM fibrosis and its effect on the hematopoietic tissue has been less studied (141). A recent study by the Balduini group (142) showed that mice constitutively expressing the EDA domain, but not EDA knockout mice, are more prone to develop BM fibrosis upon treatment with TPO. Mechanistically, EDA fibronectin binds to TLR4 and sustains progenitor cell proliferation and Mk differentiation through activation of STAT5 and ERK 1/2 signaling pathways, inducing LPS-like responses, such as release of profibrotic IL-6. Moreover, pharmacological inhibition of TLR4 or TLR4 deletion in TPO^high^ mice abolishes BM fibrosis, IL-6 release, and splenomegaly. Supporting a clinical correlate, EDA fibronectin is similarly increased in plasma and BM biopsies of PMF patients, compared with healthy controls, correlating with fibrotic phase.

Together, these studies suggest that abnormal Mk differentiation and emperipolesis are two major drivers of the release of cytokines with fibrogenic potential. However, the mechanisms underlying cytokine secretion, Mk interactions with other BM cells (e.g. neutrophils and mesenchymal stromal cells) and their functional activities in physiological conditions, as well as during myelofibrosis progression, are not yet well understood and will be an exciting subject of future studies. These could lead to new therapies aimed at disrupting abnormal Mk-BM cell interactions as a way to interfere with myelofibrosis progression.

## Concluding Remarks

Research in recent years has made substantial progress in understanding Mk development and their heterogeneous specialized functions. The changing dogma of megakaryopoiesis prompts new research areas intersecting with different fields, including immune response, cancer and stem cell biology. The ontogenic and functional relationships among Mk subpopulations are interesting areas for future studies harnessing the regenerative potential of these cells.

Emerging research suggests that Mk subpopulations exhibit specific functions beyond their roles in platelet production and hemostasis. Therefore, Mks should be studied not only as a platelet-producing cells, but also as important regulators of immune responses, HSCs and their niches. Although much has been learned recently about Mk heterogeneity, many unknowns remain regarding the signals that regulate these events through dynamic interactions of Mks with different cell types of the BM microenvironment.

Furthermore, Mk subtypes might exhibit disparate roles in steady state versus emergency hematopoiesis. The studies discussed in this Perspective demonstrate essential roles in steady state and emergency hematopoiesis, as well as in the development of malignancies, such as MPNs. However, it’s unclear whether these myriad functions are explained by distinct Mk subpopulations dynamically interacting with other cell types in the BM. These are exciting areas for future multidisciplinary research.

## Data Availability Statement

The original contributions presented in the study are included in the article/supplementary material. Further inquiries can be directed to the corresponding authors.

## Author Contributions

Both authors conceived and wrote the manuscript. EK-M prepared the figure. All authors contributed to the article and approved the submitted version.

## Funding

EK-M received outstanding postdoctoral Arab students Scholarship (from the Israeli council for higher education) and National Postdoctoral Award Program for Women in Science (from Weizmann institute of science). Original work discussed in this article was supported by core support grants from the Wellcome Trust [203151/Z/16/Z] and the MRC to the Cambridge Stem Cell Institute, National Health Service Blood and Transplant (United Kingdom), European Union’s Horizon 2020 research (ERC-2014-CoG-648765), MRC-AMED grant MR/V005421/1, a Programme Foundation Award (C61367/A26670) from Cancer Research UK to SM-F. For the purpose of Open Access, the authors have applied a CC BY public copyright license to any Author Accepted Manuscript version arising from this submission. The authors regret that some relevant literature could not be discussed because of space limitation.

## Conflict of Interest

The authors declare that the research was conducted in the absence of any commercial or financial relationships that could be construed as a potential conflict of interest.

## Publisher’s Note

All claims expressed in this article are solely those of the authors and do not necessarily represent those of their affiliated organizations, or those of the publisher, the editors and the reviewers. Any product that may be evaluated in this article, or claim that may be made by its manufacturer, is not guaranteed or endorsed by the publisher.
